# Utilisation and acceptability of formal and informal support for adolescents following self-harm before and during the first COVID-19 lockdown: results from a large-scale English schools survey

**DOI:** 10.1192/j.eurpsy.2023.1563

**Published:** 2023-07-19

**Authors:** R. Borschmann, G. Geulayov, K. Mansfield, P. A. Moran, K. Hawton, M. Fazel

**Affiliations:** 1 Centre for Mental Health, University Of Melbourne, Carlton, Australia; 2 Department of Psychiatry, University of Oxford, Oxford; 3 Department of Population Health Sciences, University of Bristol, Bristol, United Kingdom

## Abstract

**Introduction:**

Little is known about the perceived acceptability and usefulness of supports that adolescents have accessed following self-harm, especially since the onset of the COVID-19 pandemic.

**Objectives:**

We aimed to examine the utilisation and acceptability of formal, informal, and online support accessed by adolescents following self-harm before and during the pandemic.

**Methods:**

Cross-sectional survey (OxWell) of 10,560 secondary school students aged 12-18 years in the south of England. Information on self-harm, support(s) accessed after self-harm, and satisfaction with support received were obtained via a structured, self-report questionnaire. No tests for significance were conducted.

**Results:**

1,457 (12.5%) students reported having ever self-harmed and 789 (6.7%) reported self-harming during the first national lockdown. Informal sources of support were accessed by the greatest proportion of respondents (friends: 35.9%; parents: 25.0%). Formal sources of support were accessed by considerably fewer respondents (Child and Adolescent Mental Health Services: 12.1%; psychologist/ psychiatrist: 10.2%; general practitioner: 7.4%). Online support was accessed by 8.6% of respondents, and 38.3% reported accessing no support at all. Informal sources of support were rated as most helpful, followed by formal sources, and online support. Of the respondents who sought no support, 11.3% reported this as being helpful.

**Conclusions:**

More than a third of secondary school students in this sample did not seek any help following self-harm. The majority of those not seeking help did not find this to be a helpful way of coping. Further work needs to determine effective ways of overcoming barriers to help-seeking among adolescents who self-harm and improving perceived helpfulness of the supports accessed.

**Disclosure of Interest:**

None Declared

